# Books and Magazines

**Published:** 1904-07

**Authors:** 


### Books and Magazines

A TEXT-BOOK UPON THE PATHOGENIC BACTERIA.—For students of medicine and physicians. By Joseph McFarland, M. D., Professor of Pathology and Bacteriology in the Medico-Chirurgical College, Philadelphia; Pathologist to the Philadelphia Hospital and to the Medico-Chirurgical Hospital, Philadelphia. Handsome octavo volume of 629 pages, fully illustrated, a number in colors. Philadelphia, New York, London: W. B. Saunders & Company. 1903. Cloth, $3.50, net.

This work gives a concise description of the technical procedures requisite in the study of bacteriology, a brief account of the life histories of the important pathogenic bacteria, and sufficient description of the pathologic lesions accompanying micro-organismal invasions to give an idea of the origin of symptoms and the causes of death. Although but a short time has elapsed since the appearance of the previous edition, such rapid strides have been made in the subject of bacteriology, especially in its relation to pathology, that the author deemed it necessary to rewrite the work entirely. All the old matter has been eliminated, much new matter is in evidence, and, in fact, the subjects treated have been brought precisely down to date. What impressed us most were the chapters upon Infection and Immunity. All the new facts recently added to our knowledge of these subjects can here be found. The value of the work as a book of reference has been materially increased by the introduction of a large number of references to bacteriologic literature. These have been thoroughly chosen, and, in nearly all cases, give the sources of the original descriptions of the micro-organisms treated, and the important methods described. Another valuable addition is a bibliographic index containing the names of over 600 authors. Altogether, the work in its new edition is very commendable, and practitioners and students will find it of unusual value.

---

American Text-Book of Surgery.—For practitioners and students. Edited by William W. Keen, M. D., LL. D., F. R. C. S. (Hon.), Professor of the Principles of Surgery and of Clinical Surgery, Jefferson Medical College, Philadelphia; and J. William White, M. D., John Rhea Barton Professor of Surgery, University of Pennsylvania, Philadelphia. Fourth edition, thoroughly revised and greatly enlarged. Handsome octavo of 1363 pages, with 551 text-illustrations and 39 full-page plates, many in colors. Philadelphia, New York, London: W. B. Saunders & Company. 1903. Cloth, $7, net; sheep or half Morocco, $8, net.

Of the three former editions of this work nearly 40,000 copies have been disposed of. Its sale, indeed, has been the wonder of the medical publishing world. In this present edition every chapter has been extensively modified, and many of them have been partially, and some entirely, rewritten. Notably among such chapters are those on Surgical Bacteriology, Tumors, the Osseous System, Orthopedic Surgery, the Surgery of the Nerves, the Joints, the Abdomen, etc. The most recent researches of Monks on the Intestines, Crile and Cushing on Shock and Blood Pressure, Matas on Neural Infiltration and Aneurysm, Edebohls on Renal Decortications, etc., have been included. The use of paraffine in nasal deformities, the methods of spinal and local anesthesia, and the newer anesthetics have also been described. And this is but an illustration of the completeness and thoroughness of the entire work.

Besides the extensive revision and amplification of the old matter, there have been added six new chapters of the utmost importance, written by men whose positions and experience especially fit them to speak with authority. These chapters are Military Surgery, Naval Surgery, Tropical Surgery, Examination of the Blood, Immunity, and Surgery of the Pancreas. Though there was a brief chapter on the Pancreas in the third edition, in this present edition it has been expanded so greatly that it really is wholly new, the modern surgery of the Pancreas having been created since the last edition. A number of the old illustrations have been replaced by better ones, and, in addition, there have been added a number entirely new. In fact, we know of no single volume work that is even its equal in the expounding of the advanced and practical principles of modern surgery.

---

A Thesaurus of Medical Words and Phrases. By Wilfred M. Barton, M. D., Assistant to Professor of Materia Medica and Therapeutics, and Lecturer on Pharmacy, Georgetown University, Washington, D. C.; and Walter A. Wells, M. D., Demonstrator of Laryngology and Rhinology, Georgetown University, Washington, D. C. Handsome octavo of 534 pages. Philadelphia, New York, London: W. B. Saunders & Company. 1903. Flexible leather, $2.50, net; with thumb index, $3, net.

This work is the only Medical Thesaurus ever published. It performs for medical literature the same services which Roget's work has done for literature in general; that is, instead of, as an ordinary dictionary does, supplying the meaning to given words, it reverses the process, and when the meaning or idea is in the mind, it endeavors to supply the fitting term or phrase to express that idea. To obviate constant reference to a lexicon to discover the meaning of terms, brief definitions have been given before each word. As a dictionary is of service to those who need assistance in interpreting the expressed thought of others, the Thesaurus is intended to assist those who have to write or to speak to give proper expression to their own thoughts. In order to enhance the practical application of the book cross references from one caption to another have been introduced, and terms inserted under more than one caption when the nature of the term permitted. In the matter of synonyms of technical words the authors have performed for medical science a service never before attempted. Writers and speakers desiring to avoid unpleasant repetition of words will find this feature of the work of invaluable service. Indeed, this Thesaurus of medical terms and phrases will be found of inestimable value to all persons who are called upon to state or explain any subject in the technical language of medicine.

A Text-Book of Obstetrics. By J. Clarence Webster, M. D. (Edin.), F. R. C. P. E., F. R. S. E., Professor of Obstetrics and Gynecology, Rush Medical College, in affiliation with the University of Chicago; Obstetrician and Gynecologist to the Presbyterian Hospital, Chicago; Obstetrician to the Chicago Lying-In Hospital and Dispensary, Chicago, etc., etc. Handsome octavo volume of 767 pages, with 383 illustrations, 23 in colors. Philadelphia, New York, London: W. B. Saunders & Company. 1903. Cloth, $5, net; sheep or half Morocco, $6, net.

This work has been written for the student of obstetrics, as well as for the active practitioner. The anatomic changes accompanying pregnancy, labor, and the puerperium are described more fully and lucidly than in any other text-book we have seen. The exposition of these sections is based mainly upon studies of frozen specimens, in which department the author has had a larger experience than any other worker. Unusual consideration is given to embryologic and physiologic data of importance in their relation to obstetrics. The practical aspects of the subject are presented in such a manner as to be of direct assistance to the clinician. Diagnosis and treatment are presented with rare exactitude and clearness, particular consideration being given to those methods that have proved most successful by experience. Evidently great care was taken in the selection of the illustrations, aiming to meet the varied requirements of both the undergraduate and the practicing physician.

---

Clinical Examination of the Urine and Urinary Diagnosis. A Clinical Guide for the use of Practitioners and Students of Medicine and Surgery. By J. Bergen Ogden, M. D., formerly Instructor in Chemistry, Harvard University Medical School, Boston; Assistant in Clinical Pathology, Boston City Hospital, etc. Second revised edition. Handsome octavo volume of 418 pages, illustrated, including 11 plates, 9 of them in colors. Philadelphia, New York, London: W. B. Saunders & Company. 1903. Cloth, $3.00 net.

The design of this work is to present in as concise a manner as possible the chemistry of the urine and its relation to physiological processes; the diagnosis of diseases and disturbances of the kidneys and urinary passages.

The writer has foreseen the need of a treatise which takes up in detail the subject of urinary diagnosis and the application of information furnished by careful chemic and microscopic examinations of the urine.

In Part I chemic and microscopic methods are described in detail. In Part II special attention has been paid to diagnosis.

MODERN SURGERY—GENERAL AND OPERATIVE. By John Chalmers DaCosta, M. D., Professor of the Principles of Surgery and of Clinical Surgery in the Jefferson Medical College, Philadelphia. Handsome octavo volume of 1099 pages, with over 700 illustrations, some in colors. Philadelphia, New York, London: W. B. Saunders & Company. 1903. Cloth, $5.00 net; sheep or half Morocco, $6.00 net.

This work may best be characterized by being called a thoroughly up to date and strictly scientific treatise on the subject of surgery. It presents the subject in a concise manner, and reasons forward in a thoroughly practical manner from the fundamental principles of surgery. Surgery as well as all other sciences has made great strides of advancement in the last few years, and in order to keep up with the advancement of the times, the general practitioner must constantly add to his library such books as the above. The work is thoroughly revised, and much of it is entirely rewritten. Of the illustrations we desire to speak especially. Ten full-page lithographic plates, illustrating the pathological and operative features of the work, as well as its anatomy, are most excellent additions. More than two hundred other excellent illustrations on a large variety of practical points are scattered throughout its pages. We feel justified in recommending the work most sincerely.

---

A TEXT-BOOK OF DISEASES OF WOMEN. By Barton Cooke Hirst, M. D., Professor of Obstetrics in the University of Pennsylvania; Gynecologist to the Howard, the Orthopedic, and the Philadelphia Hospitals. Handsome octavo volume of 675 pages, sumptuously illustrated with some 650 illustrations, many in colors. Philadelphia, New York, London: W. B. Saunders & Co. 1903. Cloth, $5.00 net; sheep or half Morocco, $6.00 net.

The latest work of Dr. Hirst's is on the same lines as his "Text-Book of Obstetrics." As would be expected from a practical teacher, diagnosis and treatment have been given particular attention. The palliative treatment, as well as the radically operative, is fully described, enabling the general practitioner to treat many of his own patients without referring them to a specialist. A feature which specially impressed us is the thorough manner in which the author has treated modern technic of gynecic surgery. An entire section is devoted to a full description of all modern gynecologic operations, illustrated and elucidated by numerous photographs taken especiallly for this work. The author's training in the subject of diseases of women has been like that of the specialists in the Teutonic countries of Europe, where gynecology has reached the highest level of perfection; namely, specialization in the diagnosis and treatment of diseases of women has followed a thorough training in the recognition and treatment of the complications and sequels of childbirth. This special training is evident throughout the entire work in the careful and thorough manner in which the subject is treated. The many illustrations are the most magnificent we have ever seen. With but few exceptions all are entirely original, having been reproduced from photographs and water colors of actual clinical cases accumulated during the past fifteen years. We must heartily congratulate Dr. Hirst and his publishers upon the production of such a magnificent work.

---

PAIN AND ITS INDICATION. By Edward C. Hill, M. D., Professor of Chemistry and Toxicology, Denver and Gross Medical College, Chicago: G. P. Engelhard & Co., Publishers. Cloth, gilt top, $1.00.

Pain, from the standpoint of the patient, is the most important of symptoms. To recognize its cause and give relief is the first duty of the physician.

Dr. Hill has written what may be considered an "encyclopedia of pain," enabling the physician to trace this symptom to its origin and then suggesting the indicated remedy or remedies.

The work has been carefully classified, so as to present the facts is the most available form for the physician's use.

It will be found a valuable work of reference and an indispensable vade mecum in the treatment of this symptom.

It abounds in valuable prescriptions, garnered from many sources.

Treatment, as a matter of fact, occupies a very important part, it being the object of the author to point out the indications through which the cause of each and every pain can be reached, so as to effect a cure when possible, as well as to indicate the remedies of most value in each individual case, to be used for the purpose of giving relief.

---

THE PRACTICE OF OBSTETRICS DESIGNED FOR THE USE OF STUDENTS AND PRACTITIONERS OF MEDICINE. By J. Clifton Edgar, Professor of Obstetrics in Cornell University Medical College, etc. With 1221 illustrations, many of which are printed in colors. P. Blakiston's Sons & Co., Philadelphia. Price $6.00.

It is indeed with pleasure that the reviewing editor sits down to contemplate a book of this character. For wealth and adaptability of illustrations we know of no superior book, and very few equals, on any medical subject. This, which is certainly the most striking and practical feature of the book, is gone in to with a system and a fidelity of detail rarely seen. In the illustrations of the manual examinations of the female pelvic organs, the pelvic bones and ligaments are dotted so that the examining hand or instrument may be seen in its clear relation to them. This is a thoughtful and instructive feature. The relations of the intra-uterine fetus is also outlined showing its relations to the examining hand. Nearly one hundred illustrations of malformed fetuses are given, which show better than many times that space devoted to words could show deformities and monstrosities. As said before, the feature of the work is its wealth of illustration, but the book is by no means a chart. Ample text explains fully the obstetric principles involved. In fact, we can recommend the work as an exhaustive and faithful treatise on this subject.

---

Commoner Diseases of the Eye—How to Detect and How to Treat Them. By Casey A. Wood, C. M., M. D., D. C. L., Professor of Clinical Ophthalmology in the University of Illinois, etc., and Thomas A. Woodruff, M. D., C. M., L. R. C. P., Professor of Ophthalmology in the Chicago Post-Graduate Medical School, Chicago, etc. 25 illustrations; 7 colored plates. 500 pp. 5x8 in. Bound in green Buckram, gold side-title and top. $1.75 net. G. P. Engelhard & Co., Publishers.

This book will be of especial value to the country general practitioner, out of reach of a specialist. It will enable him to treat many diseases of the eye that usually seek specialists. It is intended for the general practitioner, and is well gotten up and beautifully illustrated.

---

The Practice of Medicine. A Text-Book for the Practitioner and Students, with Special Reference to Diagnosis and Treatment. By James Tyson, M. D., Professor of Medicine in the University of Pennsylvania, etc. Third edition, thoroughly revised and enlarged. One hundred and thirty-four illustrations. P. Blakiston's Sons & Co., Philadelphia.

This work of 1240 pages is a thorough and practically complete treatise of even so large a subject as practical medicine. In this the third edition we welcome the advent of a great deal of new material, especially on the subjects of malarial fever, yellow fever, gout, lithemia, the anemias and nephritis, bringing these subjects strictly up to modern ideas. The work is particularly fine on diagnosis and treatment, the two essentials in any reliable text-book on medicine. This book is unfortunately sparsely illustrated, but the pictures presented are particularly good, especially the full-page colored lithographs, representing Koplik's sign of measles, stained specimens of blood of the anemias and leukemias, and the circulation of the brain. The section on nervous diseases has been carefully revised by Dr. W. G. Spiller, and is thoroughly up to date. It gives us pleasure to endorse this book, and recommend it to our readers.

---

NOSE AND THROAT WORK FOR THE GENERAL PRACTITIONER. By George L. Richards, M. D., Fellow American Laryngological, Rhinological and Otological Society; Fellow American Otological Society; Associate Editor Annual of Otology, Laryngology and Rhinology; Otologist and Laryngologist Fall River Union Hospital, Fall River, Mass. Price $2.00. Published by International Journal of Surgery Co., New York.

The general practitioner of medicine is constantly called on either to treat such troubles or complications arising from them. He should certainly, therefore, have sufficient knowledge on the subject to be able to diagnose correctly and do a certain amount of treating. In order to keep up with this phase of his work he should have a certain number of short but practical up to date treatises on this special subject constantly at his disposal. By a careful perusal of this work we have come to the conclusion that this is an ideal work to select for a place in his library. We believe that the work, especially the treatment, is gone into with a little more detail than is necessary for one in general practice, but this is done evidently with the idea that some physicians are specializing on this subject, as well as carrying on a general practice. We believe that the little work is one of merit.

---

THE AMERICAN POCKET MEDICAL DICTIONARY. Edited by W. A. Newman Dorland, M. D., Assistant Obstetrician to the Hospital of the University of Pennsylvania. Containing the pronunciation and definition of the principal words used in medicine and kindred sciences, with 566 pages and 64 extensive tables. Fourth revised edition, greatly enlarged. Philadelphia, New York, London: W. B. Saunders & Co., 1903. Flexible leather, with gold edges, $1.00, net; with thumb index, $1.25, net.

This handy little book of six hundred pages, printed on thin paper, with flexible leather back, is indeed a little treasure in its way. Only a moment's time is necessary for finding the meaning of any word, so systematic is the arrangement. The selection of words is as complete as possible in a work of this dimension. Modern medical literature has been gleaned for all the possible new words in order to bring the work clear up to date. A good deal of matter is arranged in a tabulated form, which, likewise, facilitates hunting information. The work is indeed very cheap at the above price.

